# Does exclosure restore woody species regeneration in degraded lands? The case of Loma Bosa District of Dawuro zone, Southwestern Ethiopia

**DOI:** 10.1186/s12862-022-02067-w

**Published:** 2022-09-29

**Authors:** Assefa Ataro Ambushe, Girma Gezimu Gebre, Getahun Shanko Mamo

**Affiliations:** 1 Bureau of Agriculture, Southern Nations Nationalities and Peoples Regional State, RLLP II PROGREEN PROJECT, Hawassa, Ethiopia; 2 Faculty of Environment, Gender and Development Studies, Hawassa University, Hawassa, 05, Ethiopia; 3 The Japan Society for the Promotion of Science (JSPS) Postdoctoral Research Fellowship Program, Ritsumeikan University, Kyoto, 603-8577 Japan; 4 College of Agriculture, Wolaita Sodo University Dawuro Tarcha Campus, Tarcha, Ethiopia

**Keywords:** Exclosure, Loma Bosa District, Open degraded land, Regeneration, Woody species

## Abstract

Exclosure becomes popular as a naming of the practice of excluding degrading agents from degraded lands for natural rehabilitation. However, its role on woody species regeneration in the Loma Bosa District of the southwestern Ethiopia has not been investigated. Therefore, this study examines the role of exclosure on woody species regeneration by comparing exclosure, open woodland, and degraded land areas. A systematic transect sampling method was employed to collect vegetation data in sampling quadrats, each with a size of 20 × 20 m, evenly distributed along parallel transect lines. All the woody plant species in each plot were identified and measured for DBH and height. Twenty-six woody species, representing 16 plant families, were recorded at the study area, of which only eight were recorded all in the exclosure, open woodland and open degraded land. Species Diversity Index (H′) was 2.62, 2.38, and 1.56 for woody species in exclosure, open woodland area, and open degraded land area. Wood species density were 2225 ha^−1^, 1642 ha^−1^, and 297 ha^−1^ for exclosure, open woodland area, and open degraded land area, respectively. The distribution of the height and DBH of the recorded species in exclosure exhibited an inverted “J” shape pattern suggesting a healthy regeneration status of the important species, while others revealed irregular and less interpretable pattern. Overall results from this study indicated that exclosure is important for improvement of woody species regeneration in degraded lands in the study area.

## Introduction

Land degradation, a process of diminishing the productive potentials of land resources, is one of the serious environmental problems at the global scale [1, 2]. It has been increasing in severity and extent in many parts of the world. For instance, in 2008, the United Nations Food and Agriculture Organization (FAO) reported that more than 20% of all cultivated areas, 30% of forests, and 10% of grasslands undergoing degradation in the world. A recent study by [1] reported that about 29% of the global land area covered by the degraded land, which are affecting about 3.2 billion people who are especially rural communities, smallholder farmers, and the very poor in the developing regions of the world.

Land degradation mainly triggered by population pressure, expansion of agricultural land, deforestation, and over-exploitation of the natural resources [3–5]. Thus, combating of land degradation though rehabilitation and ecological restoration is important to ensure the long-term productivity of the land resources and survival of life on the earth [5, 6]. Closuring degraded area from human and livestock interference is one of the successful rehabilitation activities to combat land degradation and its significant negative impacts on woody species generation in developing countries [5].

In sub-Saharan Africa, land degradation is one of the biggest problems that threatening the lives of millions of people [1, 2, 7]. Like as other sub-Saharan African countries, it is one the major problems in Ethiopia. It has been negatively affecting the agricultural production, livelihoods, and provision of other ecosystem goods and services in the country.

To combat the deforestation and land degradation problems, Ethiopia has initiated extensive number of rehabilitation programs, such as exclosure, soil and water conservation activities. Particularly, the establishment of exclosure is considered as an important tool to rehabilitate the degraded land, improve agricultural productivity, restore natural vegetation, reduce soil erosion, improve hydrological cycles, and microclimate in the country [8–10].

Recently, exclosure becomes popular as a naming of the practice of excluding degrading agents (domestic grazing animals and human interference) from degraded lands for natural rehabilitation. However, several challenges are facing to make it fully realized. Establishing exclosure on degraded land is considered as a cheap and convenient means of rehabilitating degraded areas, and convenient for economically poor countries like Ethiopia. Now a day, exclosure is very common and important tool to rehabilitate degraded lands especially in the southwestern Ethiopia including Dawuro zone because of the impressive changes in terms of ecological restoration, improve ecological succession, regeneration of different plant diversity, improvement of productivity, reduction of runoff and soil erosion and over all agro-ecological stabilization. However, there is no study conducted on the role of exclosure on woody species regeneration and its species composition in Dawuro zone. Therefore, this study aims to examine the role of exclosure on woody species regeneration by comparing the woody species regeneration between exclosure, open woodland (the area covered by forest mainly plantation forest) and open degraded land area (open grazing land) in the Loma Bosa District of the Dawuro zone in Ethiopia.

## Materials and methods

### Description of the study area

**Fig. 1:**
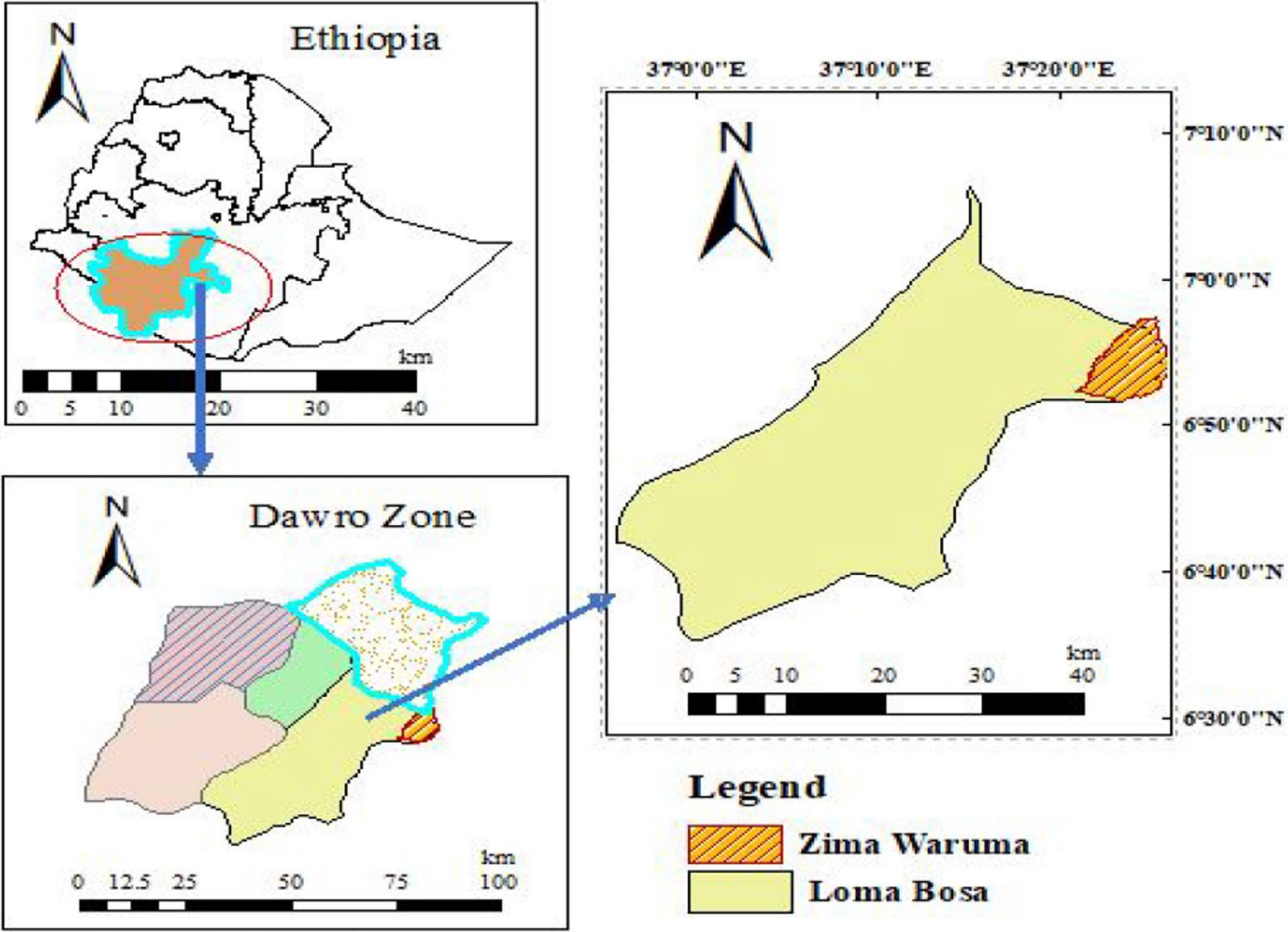
Map of study site (Source: Authors)

The study was conducted in Loma Bosa District of the Dawuro zone, southwestern Ethiopia. The geographical location lies between 6° 35′ to 7° 34′ north and 36° 04′ to 37° 53′ east [11]. For the present study, Zima Waruma was selected as shown in Fig. 1. The rationale for selecting Zima Waruma was that the practice of exclosure has been applied for many years (implemented in 2005) than other areas of the district. Though the area has ample potential for agricultural production, its farm productivity is low because farmers use traditional means of production [12] and the degradation of land resources, such as forests and soils. The population of Zima Waruma is estimated at 3724, of which 1879 are males and 1845 females [13]. To avoid edge effects, the first transect were laid 30–50 m inside the exclosures and other land use type. Before the exclusion of interference by people and livestock, the areas were under similar state of degradation, mainly communal grazing, uncontrolled removal of plants and its products by local communities and soil erosion. The open degraded areas are communal grazing lands with an open access to local community members for grazing of livestock and removal of biomass. The areas of the exclosure, open woodland and open degraded areas cover 48 ha, 51 ha, and 26 ha, respectively.

### Sampling and data collection techniques

Since this is the first investigation after establishment of the exclosure, it is challenges to explain the full process of the vegetation dynamics in study site. However, changes after the establishment of exclosure was described using some important parameters such as woody species composition (relative contribution of individual species), structure (density and cover-abundance) and diversity measurements (e.g., richness and evenness) in the study site [14] compared with the open woodland and adjacent open lands. Exclosure and other land use types selected were found within the same landscape and similar environmental conditions. The assumption in this study is that the exclosure and open areas had similar conditions before establishment of the exclosure.

#### Sampling techniques

To obtain overviews of exclosure and woody species regeneration, the reconnaissance survey was made in 2019 before the actual fieldwork in the study area in 2020. Contacts made and consent had reached with the Dawuro zone Agriculture and Natural Resource and Department of environment, forest and climate change at Loma Bosa District before starting actual fieldwork. Accordingly, senior technical experts were assigned to assist and facilitate the data collection process. Training and orientation were given to all experts involved on data collection. Moreover, a brief introduction about the purpose of the study was given for the *kebele* administrations and other stakeholders. After introduction, *kebele* leaders and development agents were identified the specific exclosure, open woodland, and open degraded land area for vegetation survey on woody species regeneration assessment.

#### Data collection method and sample size

Systematic data collection approach was used following Fikadu and Argaw [15] to determine the composition and density of woody plants in the exclosure, open woodland and open degraded land areas in the site. Parallel line transects, which have 200 m apart from each other, were laid crossing the study sites from west to east direction [16]. Along each transect, sample plot quadrats measuring 20 by 20 m (400 m^2^) were laid down at 50 m intervals. Accordingly, 30 quadrats laid in study sites and proportionally distributed to each land use based on the area coverage. Accordingly, 12 were from the each of the exclosure and open woodland areas, while the rest 6 quadrats were laid in open degraded land area near the exclosure site. In each of these quadrats, the identity and number of all individuals of woody species were determined and recorded by their local, common and scientific names [17] and nomenclatures of woody species were followed flora of Ethiopia and Eritrea [18] and useful trees and shrubs of Ethiopia [17, 19].

The diameter and height of trees, shrubs, saplings and seedlings of the woody species were measured using a diameter tape and marked measuring stick, respectively. The diameter at stump height (DSH) for shrubs and saplings and diameter measured at breast height (DBH) for trees [20]. According to Lai et al. [21], DBH and height of seedling is < 2.5 cm and < 1 m; sapling and shrub is 2.5–5 cm and 1–2 m, and tree and shrub is ≥ 5 and ≥ 2 m respectively. Regeneration status of species in study site was determined based on the population size of seedlings and saplings as good regeneration if seedlings > saplings > adults and fair regeneration if seedlings > or ≤ saplings ≤ adults [22].

### Data and analysis

The density of each woody plant per hectare was derived from the total number of individuals recorded in the total quadrats, at the exclosure, open woodland, and open degraded land areas of study site. The species diversities in all land use types (exclosure, open woodland, and open degraded land areas) of the study sites were calculated using Simpson’s Diversity Index and Shannon–Wiener Index.

The Simpson’s Diversity Index was developed by Simpson [23] and given as:

1 $$\text{D}={\sum}\left(\frac{ni(ni-1)}{N(N-1)}\right)$$

where D is Simpson’s diversity index, which ranges the value between zero and one. The zero value represents infinite diversity and one represents no diversity. That is, the bigger the value of D, the lower the diversity of tree species. n_i_ is number of individual woody species in the exclosure, open woodland, or open degraded land areas; and N is total number of woody species in the exclosure, open woodland or open degraded land areas (total number of woody species in the sample).

The Shannon–Weiner index assumes that all species are represented in a sample and that the sample was obtained randomly [24]. The index obtained from the following equation:

2 $${\text{H}}^{{\prime }}={\sum_{i=1}^R}\ln(Pi)=\ln\left(\frac1{\prod_{i=1}^RPi^{Pi}}\right)$$

where pi is the proportion of individuals that belong to species *i*; *R* is the number of species in the sample, and ln is the natural logarithm. The term in the parenthesis equal to the true diversity (i.e., *D*) and H′ = ln(*D*). A limitation for Shannon–Weiner index is that its value usually biased toward measuring species richness in a sample.

Evenness index (J) or equitability of species was calculated using the Shannon Evenness index equation as:

3 $$\text{J}=\frac{H^{\prime}}{H^{\prime}Max}=\frac{H^{\prime}}{\ln\left(R\right)}\frac{-\sum_{i=1}^R\ln(\text{pi})^{\prime}}{lnR}$$

where H′Max is equal to ln(R); H′ represents Shannon diversity index; lnR represents the natural logarithm of the total number of species in each community, and R represents the number of species in each community [24]. The higher the values of Shannon evenness (J), the more even the species are by their distribution. Likewise, the higher the value of Shannon diversity index (H′), the more diverse the community are. If the community has one species, the index will be close to zero. If all species in the data set are equally common, all *p*i values will be equal to 1/*R* and the Shannon–Weiner index equals ln(*R*). The collected data from each exclosure, based on the parameters indicated above, ware compared to its adjacent open land to evaluate the effect of exclosure on species richness and diversity. The similarity between the exclosure and open areas in their woody species vegetation was analyzed using Sørenson’s Similarity Coefficient (SSC) [25].

4 $$\varvec{SC}=\frac{2\mathbf{a}}{2\mathbf{a}+\mathbf{b}+\mathbf{c}}$$

where a represents number of plant species common to both habitats (i.e., exclosure and open areas); b represents number of species in the first habitat but absent in the second; and c represents number of species preset in the second habitat but absent in the first.

5 $$Density=\frac{\text{Total}\; \text{number}\; \text{of}\; \text{stem}\; \text{of}\; \text{all}\; \text{trees}}{\text{Sample}\; \text{size}\; \text{in}\; \text{hectare}}$$

6 $$Relative\;density=\frac{\text{Total}\; \text{number}\; \text{of}\; \text{stems}\; \text{of}\; \text{all}\; \text{trees}}{\text{Sample}\; \text{size}\; \text{in}\; \text{hectare}}\times 100$$

7 $$Frequency=\frac{\text{Number}\; \text{of}\; \text{plots}\; \text{in}\; \text{which}\; \text{a}\; \text{species}\; \text{occur}}{\text{Total}\; \text{number}\; \text{of}\; \text{plots}\; \text{laid}\; \text{out}\; \text{in}\; \text{the}\; \text{study}\; \text{site}}\times 100$$

8 $$Relative\;frequency=\frac{\text{Frequency}\; \text{of}\; \text{tree}\; \text{species}}{\text{Frequency}\; \text{of}\; \text{all}\; \text{species}}\times 100$$

The above parameters were analyzed using the PAST software package, version 4.3. Species densities, height, and diameter at breast height (DBH) were used for description of the vegetation structure. Descriptive statistics such as frequency distribution was done for the data set from the two adjacent sites using SPSS software version 21.

## Results and discussion

### Woody species composition among habitats

Out of the total 26 woody species and 16 families, 23, 22 and 8 woody species belonging to 14, 14, and 4 families were recorded in exclosure, open woodland and open degraded land areas, respectively. Of the total recorded woody species, 19 (73.1%) were common to exclosure and open woodland areas, while 8 (30.77%) were common to both habitats (exclosure and open land areas of the total sample). The results from the vegetation composition analysis indicated that exclosure areas have the richest woody vegetation composition than other habitats in the study sites. Four woody species namely *Dodonaea viscosa* (18.5%), *Combretum collinum* (18.3%), *Combretum mole* (18%), and *Dichrostachys cinerea* (16.7%) were the most dominant composition constituting 71.5% of the total woody vegetation species in the exclosures. On the other hand, *Dichrostrachys cinocera* (26.9%), *Dodonaea viscosa* (14.2%), and *Combretum collinum* (10.4%) were the most dominant composition woody species consisting 61.9% of the total woody species in the open woodland. The open degraded land is dominated by three woody species namely *Prosopis juliflora* (26.9%), *Grewia bicolar* (17.9%), and *Dichrostachys cinerea* (14.9%). Together, they consisted 59.7% of the total woody species in open degraded land (Table 1).

**Table 1:** Species composition in the exclosure, open woodland and open degraded land

No.	Scientific name of species	Family name	Exclosure		Open woodland		Open degraded land	
			Fr	RA	Fr	RA	Fr	RA
1	*Rhus ruspolii*	Anacardiaceae	3	0.3	10	1.3	0	0
2	*Uvaria leptocladon*	Annonaxeae	23	2.2	4	0.5	0	0
3	*Carissa spinarum*	Apocynaceae	13	1.3	26	3.3	0	0
4	*Vernonia ampla*	Asteraceae	3	0.3	0	0	0	0
5	Balanites aegyptiaca	Balanitaceae	0	0	10	1.3	0	0
6	*Combretum molle*	Combertaceae	192	18	70	8.9	4	6
7	*Terminalia laxiflora*	Combretaceae	80	7.5	30	3.8	6	9
8	*Combretum collinum*	Combretaceae	195	18.3	82	10.4	8	11.9
9	*Terminalia brownii*	Combretaceae	10	0.9	28	3.6	0	0
10	*Costus afer*	Costaceae	6	0.6	6	0.8	0	0
11	*Acacia seyal*	Fabaceae	0	0	2	0.3	0	0
12	*Albizia grandibracteata*	Fabaceae	58	5.4	46	5.8	4	6
13	*Albizia gummifera*	Fabaceae	10	0.9	0	0	0	0
14	*Dichrostachys cinerea*	Fabaceae	178	16.7	212	26.9	10	14.5
15	*Piliostigmli thonningii*	Fabaceae	27	2.5	26	3.3	0	0
16	*Prosopis juliflora*	Fabaceae	29	2.7	82	10.4	18	26.9
17	*Flacourtia indica*	Flacourtiaceae	2	0.2	2	0.3	0	0
18	*Pilostigma thonningii*	Legume	0	0	4	0.5	0	0
19	*Ficus glumosa*	Moraceae	2	0.2	8	1	0	0
20	*Ficus sycomorus*	Moraceae	3	0.3	0	0	0	0
21	*Dodonaea viscosa*	Sapindaceae	198	18.5	112	14.2	0	0
22	*Strychnos innocua*	Solanaceae	5	0.5	6	0.8	5	7.5
23	*Grewia bicolar*	Tiliaceae	12	1.1	2	0.3	12	17.9
24	*Grewia ferruginea*	Malvaceae	14	1.3	18	2.3	0	0
25	*Vitex doniana*	Verbenaceae	2	0.2	0	0	0	0
26	*Aloe vera*	Xanthorrhoeaceae	3	0.3	2	0.3	0	0
	Total		1068	100	788	100	67	100

Source: Field survey results

The study clearly demonstrated the importance of the exclosure for the regeneration of woody species. The results showed that the composition of woody species regeneration in exclosure area were higher than that of the open woodlands and open degraded land areas. This is due to the contribution of restriction from human and livestock interference that assisted the regeneration and succession of overall vegetation and woody species in the study site. The lower results of vegetation composition in open wood and open degraded land areas are attributes to the consequence of human and livestock interferences such as illegal cutting of trees, free or over grazing, and absence of effective keeping system. This result is consistent with the studies that concluded as humans modify the floristic composition and structure of forests during the process of utilization for their immediate purpose of best goods and services [26], but activities such as establishment of the exclosure are among other factors that assist in improving the overall ecological conditions of degraded land areas [27] and allowed regeneration of woody species.

### Species richness, diversity and evenness

The value of woody species diversity depends on the level of species richness and evenness. This study has shown species richness in the exclosure is the higher than among other corresponding habitats. The diversity value was tested in both Simpson’s and Shannon Weiner Diversity index (Table 2). The results indicated that Simpson’s diversity index (1-D), the diversity index value of exclosure, open woodland, and open degraded land areas were 0.138, 0.167, and 0.294, respectively. Thus, the lower value of (D = 0.138) for exclosure indicates the higher diversity of the woody species in a sample. The result shows that exclosure indicate that if two individuals randomly selected from a sample the probability that they belong to different woody species would be higher compared to open wood and open degraded lands. Shannon Weiner diversity index of the wood species were 2.62, 2.38, and 1.56 for exclosure, open woodland area and open degraded land area, respectively. The values indicate that relatively more unequal abundance of woody species in the exclosure than open woodland area. That is there are small number of woody species in exclosures than open woodland areas. This result is also confirmed by evenness value. The results of evenness value (J) of woody species were found to be 0.721, 0.770, and 0.751 for the exclosure, open woodland and open degraded land areas, respectively. Low evenness of woody species in exclosure reveals that the areas are dominated by a few woody species. This is because, of an illegal cutting of naturally regenerated seedling with grass, planting of a few tree species by the development program, and protection of existing shrubs and trees from illegal cutting which resulted dominance of a few woody species in the exclosure. Hence, dominance is inversely related to evenness, the exclosure are considered to be dominated by few species but with higher species richness than other habitats as shown in Table 2. The result is consistent with the studies by [15, 27, 28] that showed exclosure enhanced species richness, diversity and vegetation regeneration. This higher proportion of woody vegetation in the exclosure suggests the existence of an active regeneration and succession of woody vegetation’s. This resulted due to restriction of humans, animal interference and effective keeping system.

**Table 2:** Woody species diversity, richness, evenness density per ha in exclosure, open wood land area and open degraded land area

Habitat types	Sample (N) 400 m^2^ quadrants	Simpson’s Diversity Index (1-D)	Shannon–Weiner Diversity Index (H′)	H′ max or LN (s)	Species richness (S)	Evenness (J)	Woody species density per ha
Exclosure	12	0.138	2.62	3.135	23	0.721	2225
Open woodland	12	0.167	2.38	3.091	22	0.770	1642
Open degraded land	6	0.294	1.56	2.079	8	0.751	279

Source: Field survey results

Similarly, the species richness, diversity and density of woody species were significantly higher in the exclosure than open degraded land suggesting exclosure enhanced woody species regeneration in relatively short periods by avoiding or minimizing human and livestock interference in the degraded areas. Similar results from reported by [27–29] their studies in northern Ethiopia.

### Woody vegetation similarity

The Sorensen’s similarity coefficients of the study area were 0.844 (84.4%), 0.516 (51.6%) and 0.533 (53.3%) between exclosure and open woodland, between exclosure and open degraded land and between open woodland area and open degraded land woody vegetation similarity, respectively (Table 3).

**Table 3:** Similarity coefficient among habitat types

Habitats	Exclosure	Open woodland	Open degraded land
Exclosure	1		
Open woodland	0.844	1	
Open degraded land	0.516	0.533	1

Source: Field survey results

There is similarity of woody species regeneration across the exclosure and woodland of study sites. This similarity may be due to altitudinal range, geographic location, climatic conditions and the woody vegetation composition. There is variation of woody species composition between exclosure and open degraded and. This in turn may be due to exclosure developments, which increases the species regeneration by protecting from human and livestock interferences. The composition of woody species similarity across the sites is also not even. There is variation among exclosure, open woodland and open degraded lands, because the exclosure is supported by protection that made the rich in species composition.

### Density of woody species in life form

The density of woody species defined in this study as number of stems per hectare of all woody species in life form. The density of woody vegetation was 2225 ha^−1^, 1642 ha^−1^, and 297 ha^−1^ for exclosure, open woodland and open degraded land area, respectively. This result is line with [6, 10, 30] that the density of woody species inside the exclosure had significantly difference from open grazing land. Of the total density of all woody species, the proportion of seedling is greater than sapling, shrubs and trees in the exclosure as shown in Table 4 which is considered as successful restoration for vegetation. This is supported by findings of other study report by Fikadu and Argaw [15], who found a high proportion of seedling than others in exclosure. Total density of woody in exclosure significantly exceeds the density of their relatives in open woodland and degraded land. this replies that exclosure resulted in the best growth potential to the next generation in study site. This result is in harmony with a study by Birhane [29] who concluded that exclosure increased woody species density in Ethiopia.

**Table 4:** Density of woody species composition by life form and total density between habitats ha^−1^

Habitat	Seedlings ha^−1^	Saplings ha^−1^	Shrubs ha^− 1^	Trees ha^−1^	Total density ha^−1^
Exclosure	939.6 (42.22%)	235.4 (14.98%)	516.7 (18.82%)	543.8 (24.4%)	2225
Open woodland	377.1 (22.97%)	116.7 (7.11%)	387.5 (23.60%)	760.4 (46.32%)	1642
Open degraded land	46 (15.5%)	42 (14.1%)	83 (28%)	108 (36.4%)	297

Source: Field survey results

### Regeneration of woody species between habitats and within habitats

**Fig. 2:**
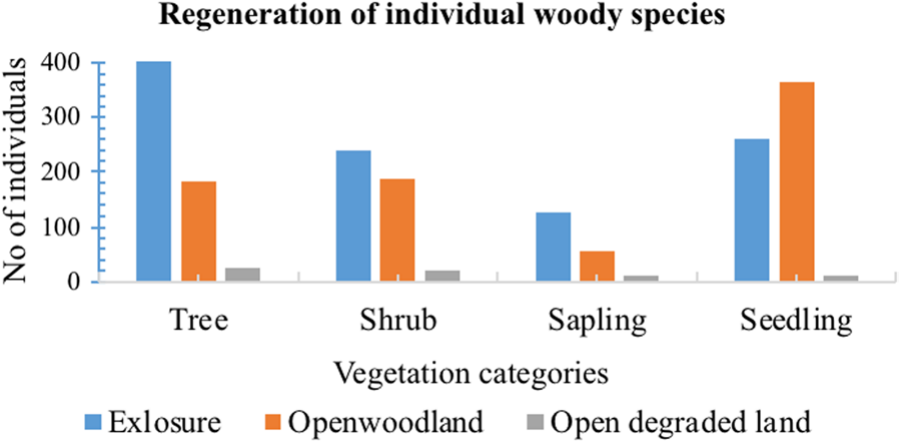
Regeneration of individual woody species at different vegetation categories

The presence of each vegetation categories like seedling, sapling, tree and shrub in all sites indicated the regeneration potential of the sites. Based on the regeneration status of 26 different woody species composition exclosure showed highest density of trees. Open woodland showed the highest seedling but less survival of sapling. The open degraded land has least density of seedling and sapling as shown in Fig. 2. This indicates that the land in the past was woodland and gradually it changed to present level of vegetation through disturbance and which negatively affected the regeneration status of the degraded land area.

### Diameter at breast height (DBH) and height of woody species

The DBH classes were divided into seven classes from DBH 0.1–5 cm to DBH 30.1–35 cm classes in three habitats. The DBH class distribution of exclosure reveled up to six classes. The DBH distribution analysis of woody species result in this habitat has shown that the DBH class constituted the majority of woody species densities per ha (compared to the other two) habitats.

**Fig. 3:**
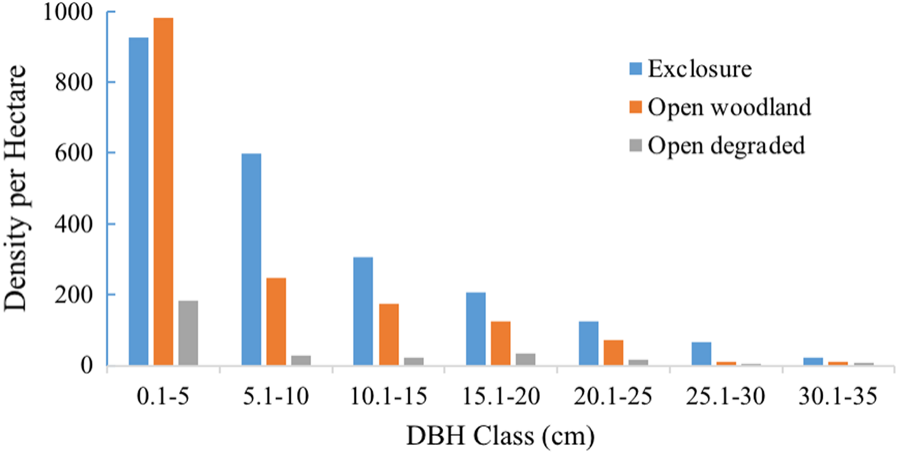
Density per hectare of woody species in DBH classes

The density of woody species in exclosure was the highest from other habitats and all DBH classes except the first class (Fig. 3). This indicates that exclosure activity enhanced woody species regeneration. Exclosure promoted species density and protection from interference improved diameter size. The pattern of DBH class distribution in exclosure revealed an inverted J-curve distribution which indicates a good potential for recruitment, reproduction and high degree of woody vegetation heterogeneity of the forest [31]. The result is in line with [32] that exclosure ensures the probability of plant growth to high diameter size which will enhance the probability of seed-bearing plants for seed dispersal and germination to seedlings which enhance future regeneration. However, drawing out of high diameter class and trees for by local people exist in study site. The predominance of small-sized individuals of the forest also shows that bigger trees were possibly removed for various purposes [33, 34]. In open woodland a sharp decline of first DBH class to the second class affected the inverted J shape slightly. However, the density distribution of vegetation in open degraded lands has not shown inverted or normal J shape pattern of distribution due to higher level of disturbance.

**Fig. 4:**
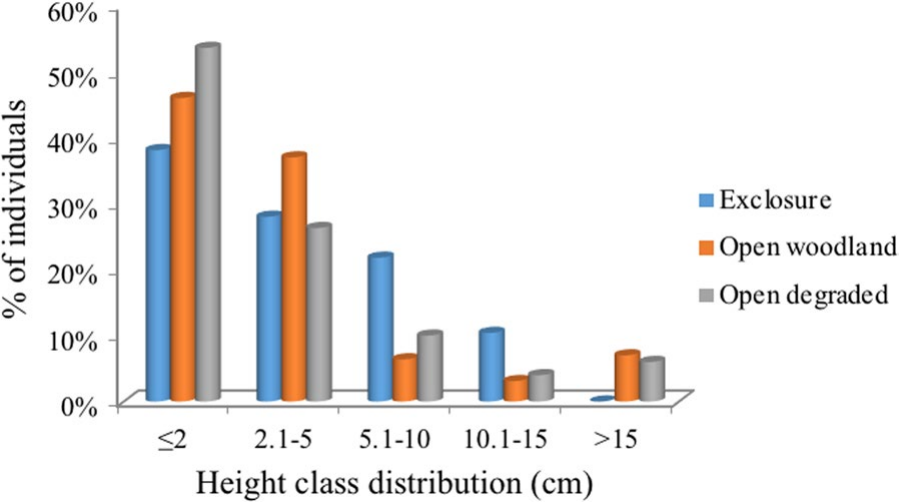
Density of woody species by height class distribution in the study habitats

Height classes were divided into five height classes from 0.1 to 2 m to above 15 m classes based on measurement results (Fig. 4). The entire seedling, saplings and some of the shrubs less than 2 m height were recorded in lower height classes in all study habitats.

The tree height analysis result has shown that the tree height% density distribution decreased with an increase in height class showing inverted J shape pattern of distribution in exclosure. This implying that the majority of the species had the highest percentage of individuals at low height classes with a gradual decrease towards high height classes. This has indicated exclosure development and management enhancing woody species regeneration and important means of solution for rehabilitation of degraded vegetation. This result is similar to the findings of other studies [35, 36] that height% density distribution decreased with an increase in height class that show healthier recruitment process and the population dynamics of the woody species under the study area. Open woodland has shown the highest number of density per ha in the lowest height class only. However, open woodland higher height class from 5.1 to 15 m trees distribution has shown much declined density and significantly affected by disturbance. This show illegal cutting of tree affected open woodland, which affect seed bearing woody species and future regeneration at risk.

## Conclusion and recommendations

### Conclusion

The research was conducted in Zima Waruma site of Loma Bossa District in Dawro zone, Southwestern Ethiopia with the aim of determining the role of exclosure on woody species regeneration through field vegetation assessment of woody species and comparing among exclosure, open woodland area and open degraded land in the study site. The results of this study showed that exclosure had the highest woody species compositions, richness, density per ha, and woody species regeneration than open woodland area and open degraded land. Exclosure showed low evenness and the highest woody species composition similarity. The exclosure had shown the higher Simpson’s and Shannon Weiner Diversity Index value than open degraded land area. The density of saplings and trees/shrubs composition in exclosure significantly exceeds the density of their counterparts in open woodland and degraded land. Exclosure showed the highest woody regeneration than corresponding open woodlands and degraded land. This indicated exclosure development could enhance woody species diversity than open degraded area. The DBH and height class in exclosure had shown the highest density distribution of woody species among other habitats in all DBH class except the first class. Exclosure showed inverted J shape pattern of woody species distribution in both DBH and height class.

### Recommendations

Exclosure should be one of the development options to solve the land and woody vegetation degradation in the study site. The effect of exclosure on woody species regeneration enhanced natural regeneration, woody species composition and richness compared to other habitats in study area. As a result, exclosure development and conservation options should be practiced at similar agro-ecological zones to sustainably manage, utilize vegetation resource in general and conserve the endangered woodland area species in particular. As a result, additional plantation with indigenous and fast-growing species, integrating soil and water conservation, water harvesting trenches and micro-basin should be introduced to improve the natural regeneration status and maximize diversity of woody species. Plantation of fast- growing multipurpose tree and shrubs, agroforestry in homestead area for household energy, construction material and forage should be considered for future sustainability of exclosure for more success of regeneration.

Finally, further detailed study is needed on the impact of exclosure on soil environment and soil seed bank, watershed development, wildlife, sustainable use of wood and non-wood products from exclosure, as well as ethno-botanical value and different uses of regenerated woody species in exclosure.

## Data Availability

The datasets used and analyzed during the current study are available from the corresponding author on reasonable request.
